# Enabling flexible integration of healthcare information using the entity-attribute-value storage model

**DOI:** 10.1186/2047-2501-1-9

**Published:** 2013-02-04

**Authors:** Dortje Löper, Meike Klettke, Ilvio Bruder, Andreas Heuer

**Affiliations:** Database Research Group, University of Rostock, 18051 Rostock, Germany

## Abstract

**Background:**

For an optimal care of patients in home healthcare, it is essential to exchange healthcare-related information with other stakeholders. Unfortunately, paper-based documentation procedures as well as the heterogeneity between information systems inhibit a well-regulated communication. Therefore, a digital patient care record is introduced to establish the foundation for integrating healthcare-related information.

**Methods:**

For the digital patient care record, suitable integration techniques are required that store data in a compact way and offer flexibility as well as robustness. For this purpose, a generic storage structure based on the entity-attribute-value (EAV) model is introduced. This storage structure fulfills the stated requirements and incoming information can be stored directly without any loss of data.

**Evaluation Results and Discussions:**

First performance tests regarding the query response time are given in this paper. The tests measured the connection time, the query execution time, and the time for traversing the result set. The time for executing the query is lowest. The time for traversing the results strongly depends on the number of documents. A concept comparison to other integration techniques is also presented.

**Conclusions:**

This approach offers flexibility concerning different standard types and the evolution in healthcare knowledge and processes. It also allows for highly sparse data to be stored in a compact way. The underlying database structure is presented, the import process for extracting incoming reports is described and the export process for generating new outgoing standardized reports is briefly illustrated.

## Background

In home healthcare, manual paperwork is still in use in several scenarios. Especially in Germany, a paper-based documentation of the care activities including some special reports is required to reside in the patient’s home and needs to be synchronized with the data in the care information system regularly. Additionally, the information is usually exchanged with other stakeholders via paper-based reports, too, and sometimes even needs to be explicitly requested.

Being involved in the healthcare-centered projects AGnES^a^[1, 2] and MARIKA^b^[3], we gathered extensive experiences in the home care domain. A home nursing service was part of the project team within both projects. Through the experiences within these projects and the close collaboration with the home care services, we gained detailed insights into the requirements for an envisioned digital patient-centered care record.

**Figure 1:**
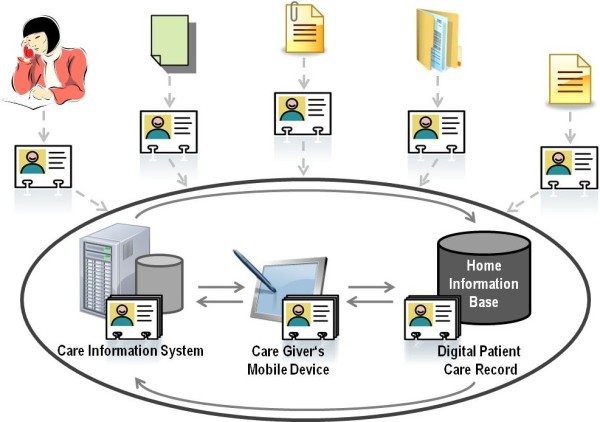
**Vision of Information Integration in Home Healthcare.** This figure shows the synchronization between the care information system in the home healthcare’s office, the mobile device that the nurse is going to be equipped with and the storage device which is supposed to reside in the patient’s home. The information exchange with other care stakeholders via standardized reports is also depicted.

Our vision is to provide the home healthcare personnel with a mobile device to support the documentation process as stated by Umblia et al. [3] and to replace the paper-based patient care record by a digital one at the patient’s home. Hence, the care information system, the mobile device and the digital care record can synchronize their patient’s information automatically. Moreover, the patient care record can be used for integrating other stakeholder’s information. In order to bypass the heterogeneity of the different stakeholder’s information systems (e.g. hospital information system, physician’s information system), standardized healthcare reports are expected to be used for exchanging data. This vision is shown in Figure 1.

An open issue is the storage structure of the digital care record. This paper focusses on the analysis of the requirements for and the subsequent development of such a storage structure. In order to include a wide variety of different information and different standards, a generic approach is necessary. Additionally, the knowledge as well as the processes are continuously developing both in the medical and in the healthcare area. Therefore, the approach should be flexible enough to handle this evolution. This paper deals with the development of a storage structure based on the entity-attribute-value model and introduces the import and export processes for this structure. It shows that the chosen storage model based on the entity-attribute-value approach is suitable for integrating diverse healthcare information. The main advantage lies in the flexibility of this approach. Moreover, the original data is kept without any changes. Only during export, the data needs to be transformed in order to fit into the designated export schema. The flexibility implicates a slight performance drawback which is also investigated in this paper.

The remainder of this article is structured as follows: This section continues with a description of the current documentation process in home healthcare, then gives an overview of the state of the art on integrating healthcare information and briefly describes some common healthcare standards. The following section “Methods” introduces the entity-attribute-value model and suggests an adequate storage structure. Moreover, the import and export processes as well as the issue of data privacy in this sensitive domain are discussed. Results of a general performance evaluation and a comparison of different concepts for information integration are presented in section “Evaluation Results and Discussions”. Finally, section “Conclusions and Future Work” concludes with a summary and an outlook.

### Current Situation of Home Healthcare Process Documentation

Currently, the care process is documented with paper-based reports. The care activities are planned with a care information system for each patient according to their needs. Preprinted forms are then taken to the patient to reside in their home. These forms are used to document the care activities and to write some special reports (e.g. about eating/drinking behavior). After a certain amount of time, the information needs to be put back into the care information system. This synchronization has to be done manually.

The format of those documents is different: some are well-structured (e.g. forms for medication, vital parameters, hygiene, treatment of wounds), others contain arbitrary texts (e.g. report about the current health status, the planned activities as well as the results).

When nurses need to exchange information, e.g. about adaptations in the care procedures for one patient, they usually do so via short paper messages or in urgent cases via a telephone call.

The information exchange with other stakeholders, e.g. physicians, other medical/healthcare experts or hospital staff, is also widely done with paper-based documents or via a (phone) conversation. For example when a patient leaves the hospital, a discharge letter is first sent to the general practitioner who then informs the home care nursing service about the changes to be made in the care routines. Sometimes, the information is not even delivered directly from the hospital staff, but needs to be requested explicitly. Some of those documents from other stakeholders have a determined report format (e.g. for patient’s transition from one care institution to another one), but they are not standardized. Sometimes this format is filled out insufficiently or it is not used at all.

Transferring information from paper to a digital format always implies additional time and effort. Furthermore, this manual work is error-prone and hence might be harmful for the patient’s healthcare. In addition to that, new information can only be accessed after a certain delay.

### Related Work on Information Integration in Healthcare

Supplying care givers with the right information in the right place at the right time is essential in home healthcare. Conflicts resulting from fragmented information can become a threat to a patient’s safety [4]. Moreover, this information should be provided automatically in order to avoid additional time and effort. In this article, an approach for a central data management is introduced. But first, an overview of existing technologies in the healthcare domain is given.

Lenz et al. [5, 6] examine the general problem context of information integration in (mainly German) healthcare networks. They describe the information systems in these networks as being highly heterogeneous and having interfaces which lack flexibility. Along with a classification of current communication standards for interoperability in healthcare, they also present a list of requirements for integrating healthcare-related information. In order to achieve more interoperability, an incremental approach is proposed and generic models are predicted to be the basis for IT systems.

Sunyaev et al. use a multi-agent architecture for integrating health information [7]. Therein, the basic medical treatment process is mapped to an agent-based technology. The key feature of the system is an active document which virtually collects requested information about one patient. However, mobile agents introduce safety hazards, which is a major drawback of this approach.

The treatment process can also be mapped to a document-based workflow [8]. While traversing the workflow elements, information units are collected from engaged institutions. Together, these information units form a resulting document representing the whole treatment process information. Both, this and the preceding approach focus on the treatment process and the composition of corresponding information. On the other hand, the digital patient record that will be presented in the article at hand expects self-contained reports.

While the two previous approaches focus on the treatment process, Haegglund et al. aim at generally integrating healthcare information from different electronic health records [9]. The data is exported from the information system into an XML file which is then mapped to an ideal XML schema and stored in a mediator database. The stakeholders access this database via mobile applications. However, due to a restricted application context, the data sources are not exchangeable. New information systems can’t be added automatically, a new mapping to the ideal XML format must be provided.

The last approach presented herein deals with a middleware platform to exchange messages between different healthcare institutions [10]. Building on different types of message standards and protocols, a client-server-architecture is implemented together with an enterprise service bus in order to separate the data transmission through designated channels from the data processing. While this approach focuses on the transfer of data, the digital patient care record could be seen as one client in the scenario.

### Standards for Health Information Communication

Standards are being developed in different areas in order to have a common concept on syntax and semantics of certain data and to ensure interoperability. In the envisioned home healthcare scenario, information from other stakeholders are expected to be received in form of standardized reports. Examples for standards which determine the structural content comprise the HL7 Clinical Document Architecture (CDA) and the CEN ENV 13606 Electronic Healthcare Record Communication (EHRcom).

Different stakeholders in the home healthcare process are interested in different parts of the overall healthcare information and deliver different information units. These may also be provided in different standards or different templates of standards. For example, the HL7 family specifies the CDA as a generic base document. With the help of implementation guidelines and templates, this generic structure can be restricted to a well-specified subarea. Examples of German implementation guides are the eArztbrief [11], a consultation note for communicating information from one physician to another, and the ePflegebericht[12] to transmit healthcare information, e.g. from a home healthcare giver to a stationary one.

As the ePflegebericht is intended to support the continuity of care, we give a brief overview of the suggested structure of this document. The head of the document is supposed to be filled with metadata of the document as well as about the patient and the stakeholders of the document. The care relevant part resides in the body of the document and is categorized into the following five main sections:

- *Nursing Process*: involves information about the patient’s care process (nursing diagnoses, interventions and outcomes)
- *Social Information*: specifies the patient’s social situation (e.g. their employments)
- *Home Care Status*: describes the patient’s current living situation (e.g. the type and character of accommodation)
- *Reference to Legal Documents*: includes information about legal guardians as well as enactments and the care level
- *Medical Information*: comprises medical diagnoses and medications

To further structure the content, several subsections are introduced to each main section. These can be used up to four levels of depth.

The authors of this transition report point out that it is only intended to deliver the necessary information in case of a patient’s transfer from one institution to another. It is not intended to be used for forwarding the whole documentation that has been produced during the care process by the first institution.

Apart from these technical standards for information communication, terminologies and classifications are used to specify uniform concepts and processes within the medical and healthcare domain. In the healthcare domain, the International Classification of Nursing Practice (ICNP) which has been developed by the International Council of Nurses (ICN) and the nursing terminology developed by the NANDA-I are examples for such classifications.

### The Digital Patient Care Record as the Main Healthcare Information Storage

The digital patient care record serves as an integration center for all healthcare information that is delivered by standardized reports from other stakeholders. At the same time, it is supposed to be the source for generating new standardized reports in order to send healthcare information to other care participants. Hence, it represents the main home healthcare information storage for a patient.

Mainly, this digital patient care record should support the home care nurses by providing the latest relevant healthcare information about a patient. Moreover, the patients themselves should be able to access their information and maybe also be informed about changes in the nurses schedule, e.g. if the nurse is late. Sometimes, physicians need to retrieve data as well, but usually they get these in forms of reports. Each of these user groups should be assigned with different access rights. Subsection “Data Security and Access Rights for the EAV Storage Solution” describes in more detail how these access rigths are realized.

The most important requirement derived from our recent project experiences with AGnES and MARIKA is that all information about one patient should be up-to-date. When the nurse carries out the care activities at the patient’s home, they must know everything that is relevant for an optimal patient-centered care. This also includes recent changes. With the current paper-based documentation, it takes some time until a necessary information, e.g. about a new medication treatment, is included in the papers. Therefore, the nurse needs to be contacted directly to be advised about the changes. This leads to a deviation in the actual up-to-date knowledge of the nurse and an outdated paper-based care record (until the care record is updated) and in case the nurse needs to refresh her knowledge she can’t consult the care record. This article mainly deals with the development of a storage structure for the envisioned digital patient care record.

Regarding one single patient, healthcare as well as medical data is highly sparse. A storage structure must be able to handle that efficiently. Additionally, it should fulfill the following requirements:

- *Flexibility*: in order to include reports that are based on different standards or standard templates
- *Stability concerning model updates*: to allow for incorporating new versions of standards or complete new standards in the future
- *Versioning*: to reproduce changes of data
- *Restorability*: the original reports have to be available
- *Traceability*: it is necessary to establish the provenience for each data
- *Extensibility*: in order to adapt to the constantly evolving knowledge and processes in healthcare

In the following section, the entity-attribute-value model will be presented as a basis for a generic storage structure for the digital patient care record.

## Methods

The entity-attribute-value model offers a generic approach for storing different kinds of information by not only storing the actual values but also reflecting the structure of the information as values in the database. The main advantage is the higher degree of flexibility concerning structural differences in the incoming information.

### The Main Concept of the Entity-Attribute-Value Model

**Figure 2:**
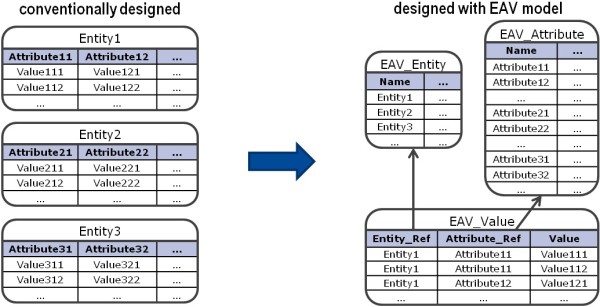
**The Main Concept of the Entity-Attribute-Value Model.** The entity-attribute-value serves for storing the structure of the information along with the actual values. This model mainly consists of three relations: The relations *Entity* and *Attribute* contain information about entities and their attributes and the relation *Value* stores the actual values for occurring entity-attribute pairs.

The entity-attribute-value model comprises three basic relations (as shown in Figure 2): the actual data are stored in the relation *value* whereas the entities and the attributes are stored in the relations *entity* and *attribute*, respectively.

The EAV model has already been used in different domains. In the health-related areas it is used to store and manage highly sparse patient data in a compact and efficient way [13–16]. The main advantages of the entity-attribute-value model are:

- *Compact storage handling*: Highly sparse data are stored in a compact way.
- *Flexible and extensible model*: This model is highly flexible, because different types of data objects can be included, even if the underlying format is different. Also, different types or implementations of standards can be supported.
- *Stability concerning model updates*: The model is also flexible regarding the evolution of data sources. New schema versions of XML-based standards or relational databases do not require any modification in the EAV structure or the already stored data. The same applies for adding new data sources.
- *Simple restoring capabilities*: No data transformation is required in order to store the incoming information, the entities, attributes and values remain the same. No data is lost. This is especially crucial in medical applications because of the patient’s sensitive data.

These features mainly cover the requirements presented in section “The Digital Patient Care Record as the Main Healthcare Information Storage”: The EAV model is flexible, updatable, extensible and the information is recoverable. For achieving traceability, the value relation(s) can store a reference to the source document. Versioning can be achieved by extending the EAV model.

On the one hand, the generic storage structure is convenient regarding input operations and the storage of heterogeneous and highly sparse data. On the other hand, the query response time is adversely affected, especially with respect to attribute-centric queries [13]. For larger amounts of data this effect increases. Thus, the entity-attribute-value model only pays off in certain applications which need flexibility. In many cases, the EAV model isn’t used exclusively but along with a conventional database schema in order to combine the advantages of both formats for different classes of data [16].

In our scenario, the data volume is relatively small, because the ambulant care givers are responsible for one patient or a restricted number of patients only. Hence, the impact on performance is expected to be relatively low.

### The Entity-Attribute-Value Concept as a Global Schema for Information Integration

The digital patient care record with the underlying EAV storage model provides the basis for integrating all care-relevant information. We extended the original EAV model by introducing separate value tables for different data types (similar to the suggestions in [16] and [17]).

**Figure 3:**
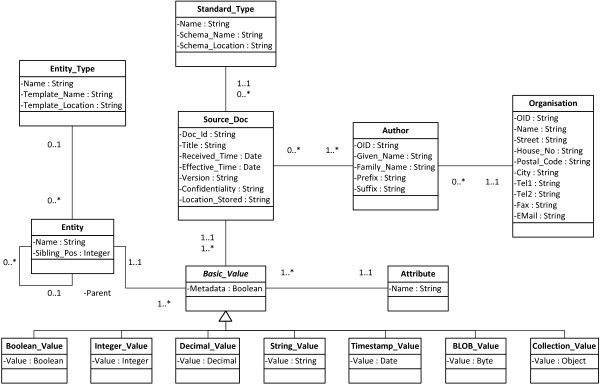
**Basic Class Model of the EAV Storage Structure.** The basic class model of the EAV storage structure extends the main EAV model by introducing separate value relations for each data type. As most of the documents to be included are XML-based, an entity’s parent and the position among its siblings are stored in the entity relation as well. The database also stores some general information about each document, e.g. information about the author.

We developed a storage structure based on the extended EAV model. Figure 3 represents the basic schema of our database storage structure. All data types that are enumerated in the ISO standard 21090 [18] can be stored in the model. For reflecting hierarchical structures (e.g. derived from XML documents), an attribute *Parent* was added in the entity relation. Moreover, an attribute *Sibling_Pos* refers to the position of an entity among its siblings.

Due to the fact that a large number of entities shares the same attributes (e.g. like in HL7 CDA), there is no direct connection between the attribute relation and the entity relation. A relationship between those tables would result in a repeated and redundant storage of the same attribute in the attribute table. The combination of entity and attribute is derived from the respective value relation instead.

Additionally, each value relation also holds foreign keys to some general information about the source document which are stored in metadata relations. These metadata comprise data about the source document including the author and possible document types and templates. Additionally, a boolean attribute *Metadata* refers to whether the data is extracted from the header part of the document (which usually contains metadata like physician’s information as well as general information about the patient and the document) or from the body part of the document (containing the actual medical or healthcare information). In the following, an example is presented which illustrates the mapping of a standardized document to the EAV storage structure. Listing 1 depicts a part of a standardized healthcare document as an instance of the German ePflegebericht, which is based on HL7 CDA (the example is taken from [5]).

### Listing 1: Extract of a Health Record based on HL7 CDA



**Figure 4:**
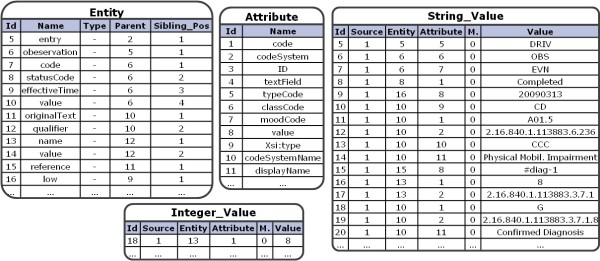
**Storing the Example in the EAV Structure.** The above example of a German ePflegebericht is stored into the shown tables. All the XML entities are stored in the relation *Entity* and their attributes are stored in the relation *Attribute*, respectively. The values are stored in the corresponding value relations. Each value tuple references one attribute and one entity tuple, respectively. A value also references the entry for the general source document information.

Figure 4 displays how this document is mapped to the EAV storage structure. For simplification purposes, only those tables are shown which contain data from the example. Importantly, all the XML elements reside in the entity relation, and the XML attributes are stored in the attribute relation, respectively. The value relations contain the actual values along with references to the corresponding attribute, entity and source_doc entry.

### Transformation Processes

In the previous sections, the integrated EAV format for storing medical and healthcare records was introduced. We initially assumed that incoming data and documents may be of different standardized forms. Furthermore, newly generated reports and documents have to comply with different standards or different versions of standards, as well. Therefore, import and export processes have to be defined.

### The Import Process

**Figure 5:**
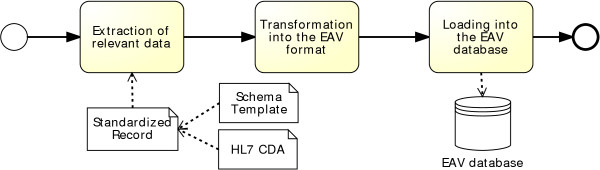
**Import Process.** The import process comprises 3 subtasks. First, the data will be extracted from the standardized report. Then, it will be transformed into the EAV format. Finally, the information is loaded into the EAV storage structure.

The import process is pretty straightforward. The subtasks that transform a document into the EAV model are shown in Figure 5. This process can be completely automated, even for new database sources or new XML document formats. Usually, no support by the domain expert is needed. All necessary information can be derived from the source databases or source XML documents. Only in case of new data formats (for instance: hierarchical databases or Excel data sheets) the import transformation component has to be extended.

The EAV model stores the original data from the sources without any information loss. Compared to other integration scenarios, that is a significant advantage, because it ensures restorability of all integrated documents.

### The Export Process

**Figure 6:**
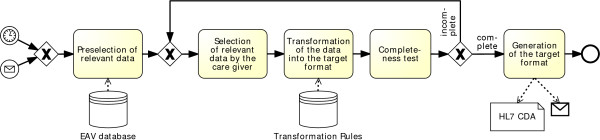
**Export Process.** The export process is divided into five subtasks. Based on some prior requirements, some preselected data will be presented to the care giver. The user selects the relevant data manually. It is then transformed into the target format with the help of transformation rules which still need to be defined. It is then checked whether the document is complete. If not, the care giver checks the selected data again and modifies it. If the document is complete, the target format is generated.

Exporting data from the EAV model is more complicated, though. We have to overcome the heterogeneities introduced by the different data sources. Figure 6 shows the subtasks for the export process. In most cases, newly generated reports only contain a fraction of the stored patient data. The selection of the data to be exported can be divided into two steps: The care giver is provided with a preselected list of data, e.g. which are shown on a mobile device, and then chooses the relevant data among this list manually. The most complicated task within the export process is the *Transformation*. For this task, transformation rules are applied for the mapping of data, which are either prepared (available in the system for known data formats) or have to be extended by the domain expert for new and previously unknown data formats.

The next subtask is a test of *Completeness* where the system checks whether the generated documents are valid and contain all necessary data. Otherwise, for instance if not all personal data of a patient are contained in the document, then the whole export process is repeated and restarts with the data selection. This process is performed until a valid export document is generated. If new databases, databases with evolved structure or documents with unknown XML schemas are integrated into the system, the transformation needs to be extended.

### Data Security and Access Rights for the EAV Storage Solution

In our digital patient care record, sensitive data about a patient are collected and stored in a database. Protecting this sensitive medical data is absolutely essential. The storage solution has to ensure that only authorized persons and user groups can change or access the data. Therefore individual rights for specific users or groups have to be set properly to allow access to well-specified portions of the data. The EAV storage solution stores all values in the same relation. Because database management systems can specify access rights for users only on complete tables, the view concept of databases has to be applied. Views can be defined to select subsets of data from the original EAV database. Database systems allow to specify access rights for reading, adding, deleting and updating data for each view separately and subsequently for the corresponding fractions of data. Accordingly, views can be used to assign access rights for each user group. Hence, horizontal and vertical fragments of the original database along with the corresponding access rights can be specified. For instance, it is possible to select only data of the previous day or the prescribed medication of a patient. Thus, the sensitive data within the EAV storage solution is protected.

## Evaluation Results and Discussions

This section presents some evaluation results for querying the data in the entity-attribute-value storage system. We implemented our EAV storage system using Oracle Database 11.2.0 and Java SE 6. Currently, we are inserting all values into the *String_Value* table. For evaluating the query response time, we chose two different kinds of queries:

1. One general query which basically returns all entity-attribute-value tuples in the database and
2. One attribute-centric query which returns all entity-attribute-value tuples that comprise those documents where a given search term was found in.

To reconstruct the exact position of an entity in the document, the ancestors and their position among the siblings need to be considered. Therefore, we use a Common Table Expression within a Materialized View to recursively compose the whole ancestor path for an entity. The entity path is composed of the ancestors’ names along with their sibling position. An example of one resulting tuple in the form of {entity path, attribute, value} is given below:

For the first query, we ran a time measurement test five times with two different scenarios: with 10 documents inserted (twice) and with 100 documents inserted (three times). Each test consisted of ten consecutive runs. The results are shown in Table 1. We split up the time measured into three sections: *Before Querying* which mainly includes getting the connection to the database, *Querying* which represents the actual query execution time and *After Querying* which mainly consists of traversing the result set. Due to the caching mechanisms of the database system, the last nine runs of each test were significantly faster in the first two sections than the first run.^d^ Hence, the first run as well as the average of all the ten runs in one test are shown.

**Table 1:** **Evaluation Results for the First Query (Time Measured in Milliseconds)**

	Before Querying		Querying		After Querying	
**Test No**	**First Run**	**Average**	**First Run**	**Average**	**First Run**	**Average**
1 (10 docs)	478	464.5	15	17	5	4.1
2 (10 docs)	488	126.1	18	4.1	3	1.1
3 (100 docs)	452	122.8	29	4.3	2745	2624
4 (100 docs)	479	124.6	27	4	2693	2588.8
5 (100 docs)	566	152.8	18	3.1	2987	2855.4

Table 1 presents the results of the query response time evaluation for the first query. This first query basically returns all the entity-attribute-value tuples from the database. It was executed in two different scenarios: with 10 documents inserted and with 100 documents inserted, respectively. The time measured was split up into three sections: *Before Querying*, *Querying* and *After Querying*. Additionally to the average time measured of ten consecutive runs, the time for the first of those ten runs is also presented as it is the only time not affected by the caching mechanisms of the database system. The focus lies on the actual query execution time which is by far the lowest compared to the time before and after querying in both scenarios.

With only 10 documents in the database, most of the time measured was used before querying with at most 500 ms. The time measured for querying was below 20 ms. Compared to that, the main impact in the scenario with 100 documents can be seen in the performance after querying. The time measured was around 3 seconds which is due to the amount of tuples (39,584) that had to be traversed within the result set. However, we don’t expect queries to deliver this amount of tuples in the real application scenario. The performance before querying was similar to the one in the scenario with 10 documents. The time measured for the actual query was between 18 and 29 ms in the first run and therefore at most only 50% higher than in the scenario with 10 documents.

For the second query, we used two different search terms to query the EAV data of 129 documents in the database. The first search term ”hemoglobin” is present in 9 of these documents, the second search term ”medication” can be found in 30 documents. The query was constructed by using a subquery which delivers all those source document ids where the search term occurs in the source document. The actual query is similar to the query above, only extended by a selection: it returns all the entity-attribute-value tuples which belong to those source documents found by the subquery.

For each of those two search terms mentioned above, we conducted four tests with ten consecutive runs. The same effect as for the first query appeared: due to the caching mechanism of the database system, the second to tenth run of each test was faster than the first one. Therefore, we summarized the results for each search term in Table 2. Again the time measured was split up into the time *Before Querying*, *Querying* and *After Querying*. For each of those three sections, the average as well as the minimum and maximum amount of time measured of the 40 test runs for each search term are displayed in the table.

**Table 2:** **Evaluation Results for the Second Query (Time Measured in Milliseconds)**

	Before Querying			Querying			After Querying		
**Query No**	**Min**	**Avg**	**Max**	**Min**	**Avg**	**Max**	**Min**	**Avg**	**Max**
1 (”hemoglobin”)	79	132.4	578	46	56.5	109	171	200.9	312
2 (”medication”)	71	130.6	514	46	58.1	109	280	313.2	484
Overall	71	131.5	578	46	57.3	109	171	257.1	484

Table 2 presents the results of the query response time evaluation for the second query. This query is attribute-centric and returns all those entity-attribute-value tuples which are part of those documents which contain a specific search term. The query was executed in four tests with ten consecutive runs for two different search terms on the entity-attribute-value data of 129 documents inserted. Hence, for each search term, the query was executed 40 times. Again, the time measured was split up into three sections: *Before Querying*, *Querying* and *After Querying*. The table displays the average as well as the minimum and maximum values of the 40 runs for each search term separately. As for the results of the general query, the time for actually querying the data is the lowest.

The results for the two different search terms are similar. When focusing on the maximum values for the three measurement sections, most time was spent before querying: about half a second. The actual querying process took about one tenth of a second and was independent from the search term. Only the results for the time measured after querying differ, which is due to the fact that the second search term occurred in three times more documents than the first one and therefore delivered a larger amount of tuples.

We conclude that the time measured for executing the actual query is the lowest compared to the measurements before and after the query. Especially for the first query, the maximum time measured is below 30 ms (with a minor increases with the number of documents). Compared to that, the query execution time for the second query was about four times slower (based on the maximum values). As expected, this attribute-centric query takes more time to execute, but this performance decrease is still acceptable. The time needed before querying is independent from the query and the number of documents in the database.

These results only reflect one query for getting all tuples and one simple attribute-centric query. An evaluation with more complex attribute-centric queries is part of our future work. Although we know that these queries are less efficient on EAV structured data, the results of the first evaluation are promising.

Another interesting point would be to compare the entity-attribute-value approach with other data integration techniques. Many data integration techniques use a middleware or a mediator to provide an integrated, global data schema. The data can be integrated permanently into this global schema (materialized integration) or the queries against the global schema will be translated into queries for the local schemas (virtual integration). Another common technique is using a query language which can address different subsystems (integration without additional layer). Table 3 discusses the differences between EAV and the other mentioned data integration techniques regarding the design costs for the integration system as well as for the querying, the query performance, the memory usage and the handling of new or altered schemas.The main difference of the EAV approach compared to the other integration concepts is the proportion between the efforts to integrate the data vs. the efforts to query the data. With the EAV approach, the actual integration is quite simple, but designing and executing the queries is more complex. However, the main advantage of the EAV approach remains the flexibility concerning new information and new data sources as well as the fact that no data is lost during integration.

**Table 3:** **Comparison of Different Integration Techniques with the EAV Approach**

	Information Integration Concepts			
**Properties**	**materialized integration,**	**virtual integration,**	**w/o integration layer, e.g.**	**direct storage of integrated data,**
	**e.g. middleware with global schema**	**e.g. mediator-based**	**a schema query language**	**e.g. EAV**
Design Costs for	high, global schema and the mapping	high, definition of global	the integration will be designed by	low, EAV can store structured documents
Integration System	between local and global schemas	schema and the mapping	user during the query definition	without designing a schema mapping
	needs to be developed	between local and global schemas		
Design Costs for Queries	low, common query	may have problems with	high, the subsystem and integration	high, the integration possibilities have to
	techniques possible	heterogenious subsystems	possibilities have to be defined manually	be defined within the queries
Handling of new or	new transformation to	new mediator required	schema queries need to be altered	no problem with new or altered schemas
altered schemas	global schema needed			
Memory Costs	high, all data is stored in	low, no data is stored within	low, there is no integration layer	high, similar to materialized integration
	a global database	integration layer		
	in the middleware layer			
Query Performance	high, only the global system	low, due to query translation	the location of the data has to be defined	there might be many operations needed
	needs to be queried	and distributed query processing	in the query, depends on subsystems	for answering complex queries

Table 3 presents a comparison of concepts for integrating information. Materialized integration refers to implementing a middleware with a global schema which all the local schemas of the data sources need to be mapped to. A virtual integration is usually based on mediators and wrappers which are introduced for each data source. Using a schema query language as a meta language to query a number of different data sources at the same time is one example for an integration without an additional integration layer. The entity-attribute-value model is a way of directly storing the integrated data. Those four concepts are compared regarding their design costs for both, the development of the global integration system and the queries against it. Moreover, the handling of new or altered schemas is evaluated and the costs for memory usage as well as the query performance are analyzed.

## Conclusions and Future Work

In home healthcare, it is very important that information is provided instantly. Current systems cannot assure this requirement, because many processes in healthcare are still paper-based. The heterogeneity of the available systems, i.e., a lack of interoperability, inhibits a fast and flexible exchange of information.

The common standards for exchanging documents in healthcare are rather generic. Therefore, an adequate storage structure should also be generic in order to handle the heterogeneous data. In this paper, a fundamental database schema based on the entity-attribute-value paradigm is introduced. A mapping of medical documents to this schema can be simply obtained by extracting particular information from the document. On the other hand, generating standardized documents from the data in the database is considered to be more difficult.

Many other applications use the EAV model for a uniform representation of heterogeneous data. Some of them only offer a search functionality for the data stored in the EAV model. Our approach adds another functionality. By offering an export service, we transform the data in the EAV database into different target formats. Thus, the EAV model is used as the global schema of a heterogeneous federated database architecture. The EAV database is mainly appropriate for this task because of its characteristics: it is able to handle heterogeneous data, and at the same time it allows for future extensions.

The flexibility and dynamic of the entity-attribute-value approach is an important advantage. However, the disadvantage is a possible performance problem at the query processing. A first evaluation showed positive results regarding the performance time for a general query and a simple attribute-centric query. Further investigations need to be conducted with more complex attribute-centric queries, though. Nevertheless, we can expect the amount of patient data to be relatively small within our scenario. Hence, we expect the impact of the more complex attribute-centric queries on performance to be relatively low. Another method to possibly increase the performance is to introduce special index structures, which should also be investigated.

The future work also comprises the definition of views including the development of a role concept for accessing the stored information. Furthermore, the described export transformation process needs to be implemented. The complexity of this process arises from the definition of transformation rules to map the data from the entity-attribute-value structure into the required information in the target format.

## Endnotes

^a^AGnES: project for systemic assistance and intervention for community primary care with the help of e-health technologies in order to relieve general practitioners (german: Arztentlastende, Gemeinde-nahe, E-Healthgestuetzte, Systemische Intervention)^b^MARIKA: project for assisting the home care nurse by supporting the documentation process and the route management with a mobile device (german: Mobile Assistenzsysteme fuer RoutenInformation und KrankenAkte)^c^ This query will later be refined to deliver only those tuples from a given document that the current user is allowed to see. Here, another advantage of the EAV storage model is shown: user rights can be set on a finer grained level than just on a whole document.^d^ Surprisingly, the caching mechanism didn’t seem to influence any run of the first test. However, the following analysis focuses on the worst case scenario for caching: the first runs. Therefore, this special occurrence was not investigated further.
